# The causal impact of older siblings’ academic achievement on younger siblings’ risk for drug use disorder: instrumental variable and propensity score analyses

**DOI:** 10.1017/S0033291726103729

**Published:** 2026-03-27

**Authors:** Kenneth S. Kendler, Henrik Ohlsson, Abigail A. Fagan, Jan Sundquist, Kristina Sundquist

**Affiliations:** 1 Virginia Institute for Psychiatric and Behavioral Genetics, Virginia Commonwealth University, Richmond, VA, USA; 2 Department of Psychiatry, Virginia Commonwealth University, Richmond, VA, USA; 3 Center for Primary Health Care Research, Sweden; 4 Department of Sociology and Criminology & Law, University of Florida, Gainesville, FL, USA; 5 Center for Primary Health Care Research, Department of Clinical Sciences, Lund University, Malmö, Sweden; 6 University Clinic Primary Care, Skåne University Hospital, Region Skåne, Sweden

**Keywords:** drug use disorder, academic achievement, genetic risk, adolescence

## Abstract

**Background:**

Drug use disorder (DUD) clusters in families due partly to shared environment, including sibling influences. Low academic achievement (AA) in adolescence increases DUD risk. This study examined whether low AA in an older sibling causally increases DUD risk in younger siblings.

**Methods:**

We studied all Swedish full sibling pairs (*n* = 309,666) born 1972–1985 and ≤ 5 years apart. Older sibling AA was assessed at age 16. Using Month-of-Birth (MoB) as an instrument, we conducted instrumental variable (IV) analyses and propensity score (PS) models to evaluate the causal impact of older sibling AA on younger sibling DUD risk, assessed by DUD registration in national medical, criminal, or pharmacy registries.

**Results:**

Older sibling AA significantly predicted younger sibling DUD risk across models. Beta coefficients (±95% CI) were 2.04 (1.97–2.12) in raw analysis, 1.88 (0.74–3.02) in IV, and 1.26 (1.17–1.34) in PS models. Together with the strong first-stage association, the IV estimates remain positive under small departures from the ideal identifying assumptions. Effect sizes declined with increasing sibling age differences (*p* = 0.036 for IV; *p* < 0.0001 for PS) and were strongest in male–male pairs (IV: 4.01 [1.42–6.61]; PS: 1.74 [1.55–1.93]). Mediation by older sibling DUD was modest.

**Conclusions:**

Findings from two causal inference approaches support a largely causal link between low AA in an older sibling and increased DUD risk in younger siblings. Stronger effects in close-aged and male–male pairs further support this conclusion. Interventions to improve AA in older siblings may yield indirect preventive benefits for younger siblings.

Drug use disorder (DUD) runs within families (Merikangas, Li, et al., 2009; Merikangas, Stolar, et al., 1998) and twin and adoption studies often suggest that this familial resemblance results from both genetic and familial–environmental factors (Cadoret, Troughton, O’Gorman, & Heywood, 1986; Kendler et al., 2013; Kendler, Sundquist, Ohlsson, et al., 2012; Maes, Neale, Ohlsson, et al., 2016; Tsuang, Lyons, Eisen, et al., 1996; van den Bree, Johnson, Neale, & Pickens, 1998). Using technological developments in molecular genetics, a major effort is underway to clarify the biological pathways that underlie this genetic transmission. Clarifying causal environmental pathways within families is inherently more challenging.

Although parent/child similarity is often the focus of such research, empirical literature has also demonstrated substantial correlations in sibling substance use and DUD (Feinberg, Solmeyer, & McHale, 2012; Rowe & Gulley, 1992; Wakefield & Baker, 2024)and consistently finds significant correlations between drug use in older and younger sibs. While genetic transmission is acknowledged, so is the likelihood that siblings directly influence each other’s substance use behavior via a ‘social contagion’ process (Altonji, Cattan, & Ware, 2017; Brook, Brook, & Whiteman, 1999; Brook, Whiteman, Brook, & Gordon, 1991; Brook, Whiteman, Gordon, & Brenden, 1983; Kothari, Sorenson, Bank, & Snyder, 2014; Needle et al., 1986; Parental, 2000; Rende, Slomkowski, Lloyd-Richardson, & Niaura, 2005; Rowe & Gulley, 1992). Consistent with this literature, we have shown that DUD in Swedish sibling pairs is linked to their geographical proximity, as well as their similarity in age and sex (Kendler et al., 2019). Convincing evidence of environmental transmission of DUD between siblings has also been shown in clinical trials demonstrating spillover effects; i.e. reductions in problem behaviors for one sibling when the other has been the focus of treatment (Caspi, Lardier, & Barrios, 2018; Rowland, Chapman, & Henggeler, 2008). For example, results from a randomized trial of a therapeutic family therapy program for drug-abusing adolescents in juvenile drug courts found that rates of illicit drug use declined significantly among the participants as well as among their 70 teenage siblings (Rowland et al., 2008).

The specific mechanisms involved in the social contagion of DUD from one sibling to another are not fully understood and are the focus of this study. Prior work suggests that sibling resemblance occurs because children share the same familial risk factors for DUD (e.g. low income, lack of parental monitoring), are both exposed to the same peer risk factors (e.g. peers who use illicit substances), and/or through modeling and imitation of drug use or related antisocial behaviors) (Rende et al., 2005; Wakefield & Baker, 2024).

In this paper, we seek to expand the range of possible mechanisms for sib-to-sib transmission of DUD risk that can be examined empirically. Prior evidence shows that poor academic achievement (AA) in adolescence is strongly predictive of increased rates of risk of drug use and subsequent DUD (Fothergill et al., 2008; Gauffin et al., 2013; Schulenberg, Bachman, O’Malley, & Johnston, 1994) and that, based on Swedish population data, low AA in high school strongly predicts DUD risk and this effect is likely to be largely causal (Kendler et al., 2018). Students who do well academically are prone to develop positive attitudes toward school as an associated prosocial lifestyle that lower risk for DUD (Hirschi, 1969). Students who do poorly in school may not view academic work positively and are more prone to deviant behaviors, including DUD (Catalano & Hawkins, 1996).

In addition, similar to the transmission of DUD, there is evidence for sibling spillover effects in which an older siblings’ AA independently influences the AA of his/her younger siblings (Altonji et al., 2017; Hauser & Wong, 1989; Karbownik & Özek, 2023; Nicoletti & Rabe, 2019). This paper builds on these prior findings to determine whether low AA in an older sibling increases risk for DUD in a younger sibling.

To address the nature of the association between AA in the older sibling and the long-term risk of DUD in the younger sibling, as outlined in Figure 1, we first conducted instrumental variable (IV) analysis (Boef, Souverein, Vandenbroucke, et al., 2016; Tchetgen Tchetgen et al., 2015) which requires us to identify an “instrument,” a variable that influences our key risk factor – here AA in the older sibling – but has no direct impact on our outcome – here DUD in the younger sibling. Given the difficulties of causal inference in non-experiment research designs, we then try to confirm results from our IV analyses with a causal inference method based on entirely different assumptions: Propensity Score Analysis (PSA) (Austin, 2011).

**Figure 1.**
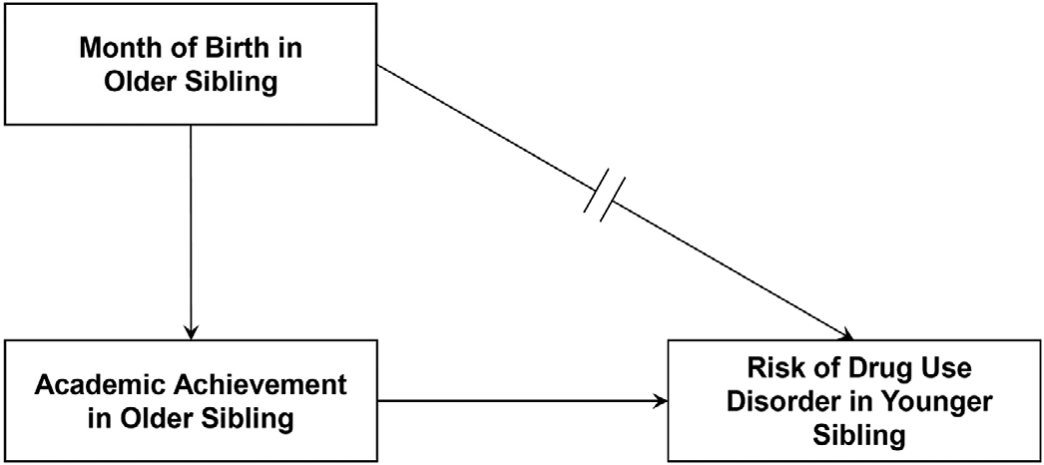
A schematic instrumental variable design. The key features of this design are illustrated which are that the instrument – here, month of birth in the older sibling – has a direct effect on the outcome -- here, risk for Drug Use Disorder in the younger sibling – only through the risk factor – academic achievement in the older sibling. The instrument must not, as illustrated, have any appreciable direct influence on the outcome.

In this paper, we seek to address four questions:

First, can we show, using standard regression methods, evidence for an association between poor AA in an older sibling and higher DUD risk in his/her younger sibling?

Second, using both IV and PSA methods, can we determine the degree to which this association is likely causal versus the result of confounding variables?

Third, does this association vary as a function of the sibling age-difference and the sex composition and the mean level of DUD genetic risk of the sibling pair?

Finally, we explore whether the association between AA in the older sibling and risk for DUD in the younger sibling may be explained by older sibling’s own DUD or other correlated behaviors.

## Methods

We analyzed individual-level data from Swedish population-based registers with nationwide coverage (see Appendix Table 1). These registers were linked using each individual’s unique identification number, which was replaced by a serial number to maintain confidentiality. Ethical approval for the study was obtained from the Regional Ethical Review Board at Lund University. From these registers, we identified all full sibling pairs born 1972–1985 with an age difference of ≤ five years. Both siblings had to be registered in the National School Registry with available AA data at age 16 and reside in the same household for more than 80% of the time until the older sibling reached age 16. We selected only one younger sibling per older sibling (proband), choosing the one closest in age, thereby identifying 309,666 full-sibling pairs. The National School Registry contains grade point average data for all students in grade nine. In Sweden, education is mandatory from ages 7 to 16. Between 1988 and 1997, grades were reported on a 1–5 scale and assessed using a peer-referencing system, resulting in normally distributed grades with minimal inflation over time. From 1998 onward, grades were reported on a 10–320 scale and based on a criterion-referenced system that assessed students’ mastery of defined competencies. These scores were not standardized across schools and were not normally distributed. To ensure comparability across cohorts, we standardized grades (mean = 0, SD = 1) by year and sex, and reverse-coded them so that higher values represented lower AA in all models. Data also included lifetime registration for drug use disorder (DUD) in both the proband and the sibling through 12/31/2018. A detailed definition of DUD is provided in Appendix Table 2.

**Figure 2.**
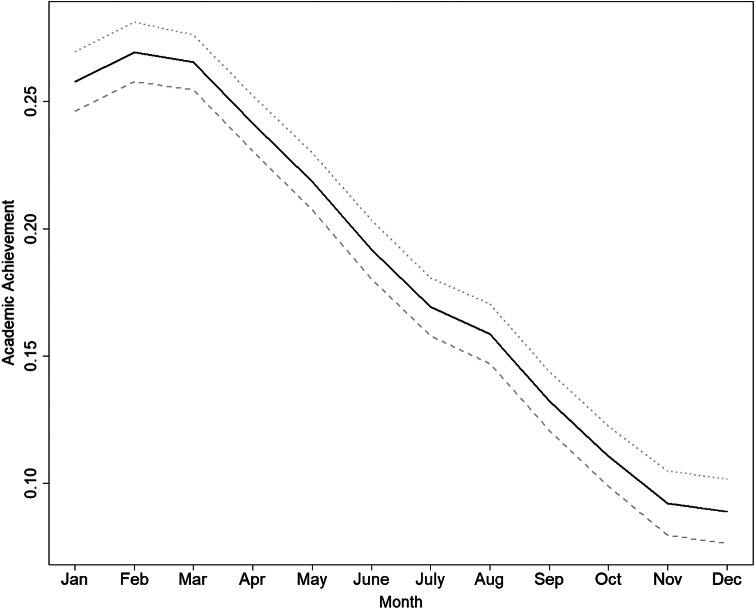
Month of birth and academic achievement in the 309,666 first-born older siblings in the sample. The *Y* axis is standardized academic achievement across the entire Swedish population. The *X*-axis is the sibling’s month of birth. The distribution of academic achievement in Sweden is shifted upward in first-born children.

To investigate the association between AA in the proband and DUD registration in the younger sibling, we first conducted ordinary least squares (OLS) regression. The resulting beta coefficients represent the percentage increase in the risk of sibling DUD for each one-standard-deviation increase in the proband’s AA. We also present covariate-adjusted regression estimates including the same set of covariates as used in the propensity score model (Appendix Table 4), with confounder selection performed using lasso regression. LASSO performs both shrinkage and variable selection, making it suitable for high-dimensional data. We then applied an IV approach to address unmeasured confounding. We used the month of birth (MoB) as an IV for the regressor (AA in the proband), under the assumption that any effect of MoB on DUD operates primarily through AA. As seen in Figure 2, the association between MoB and AA in the proband was −0.0186 SD per month (95% CI: −0.0196 to −0.0176), indicating that grades decrease with later birth months. Instrument strength was evaluated using the first-stage *F*-statistic, and the Wu–Hausman test was used to assess whether the IV estimates differed meaningfully from the ordinary regression estimates. Some concerns have been raised regarding the use of MoB as an instrument, as parental characteristics (e.g. educational attainment) may vary systematically by child’s MoB. Such variation could violate the IV assumption. To address this, we conducted sensitivity analyses controlling for parental education. We also conducted exploratory analyses that additionally adjusted for DUD registration in the proband to examine how estimates change when accounting for a potential pathway operating through the proband’s own DUD. To further evaluate the plausibility of the IV assumptions, we examined whether the older sibling’s MoB was associated with observed covariates included in the propensity score model (Appendix Table 5). For each covariate, the table reports: (1) its association with the instrument, (2) the corresponding *p*-value, (3) the MoB–AA association from the first stage when adjusting for that covariate, and (4) the IV estimate of AA on DUD in the younger sibling when the covariate is included in both stages. Covariates showing evidence of association with MoB are highlighted. Finally, we conducted analyses allowing for small direct effects of MoB on the outcome using the Conley–Hansen–Rossi Union-of-Confidence-Intervals approach.

Despite the time-to-event nature of our data, we use a two-stage least squares (2SLS) model to estimate the causal effect, primarily due to the computational simplicity and well-established inference procedure. These benefits, along with the ease of interpretability, result in the use of linear models as an accepted approach in IV contexts (Baiocchi, Cheng, & Small, 2014).

In a secondary analysis, we compared the IV results with those from a propensity score-based approach. Using LASSO (Least Absolute Shrinkage and Selection Operator) regression, we predicted AA in the proband based on a set of 35 provided covariates selected from multiple Swedish registries (see Appendix Table 3 for details). The predicted values from the final LASSO model were then included as a covariate in a crude model of sibling DUD, where the actual AA in the proband served as the exposure. This setup allows us to compare individuals with identical predicted AA but differing actual performance. All statistical analyses were conducted using SAS 9.4 (SAS Institute Inc, 2012).

## Results

The features of our sample of 309,666 older and younger full sibling pairs are seen in Table 1. On average, the sibling pairs are slightly less than three years difference in age. Lifetime risk for DUD in the siblings is between 3 and 4%.

**Table 1.** Features of the 309,666 older and younger full sibling pairs

	Older sibling	Younger sibling
Mean (SD) year of birth	1977 (3.1)	1980 (3.2)
% Males	51.1%	51.6%
Mean standardized academic achievement	−0.19 (1.0)	−0.08 (1.0)
Lifetime prevalence of drug use disorder	3.32%	4.28%
Mean (SD) of age difference	2.93 (1.0)	

Table 2 presents the results of the three regression models. A standard additive regression model showed that for each SD decrease in the older sibling’s AA, the increased risk for lifetime DUD in the younger sibling (with 95% CIs) was relatively precisely known and substantial: 2.04% (1.97–2.12). After covariate adjustment, the estimate was attenuated to 1.62% (95% CI: 1.54–1.70). In our instrumental variable analysis, the increase in DUD risk in the younger sibling was slightly less than the crude estimate – 1.88% (0.74–3.02) – with considerably larger CIs. The results of the propensity score analyses yielded a lower estimate of the increased DUD risk younger siblings: 1.26 (1.17–1.34) but with much tighter CIs.

**Table 2.** Results from the standard, instrumental variable and propensity score regression models predicting risk for drug use disorder in the younger sibling from academic achievement in the older sibling

Type of regression	Additive prediction of risk for drug use disorder in younger siblings from academic achievement in older sibling		
	Standard	Instrumental variable	Propensity score
Prediction of risk	2.04 (1.97; 2.12)	1.88 (0.74: 3.02)	1.26 (1.17; 1.34)
Age difference (interaction)	<0.0001	0.0362	<0.0001
1 y	2.59 (2.43; 2.76)	4.13 (1.62; 6.64)	1.71 (1.54; 1.88)
2 y	2.30 (2.20; 2.40)	2.88 (1.32; 4.45)	1.48 (1.37; 1.58)
3 y	2.01 (1.94; 2.09)	1.64 (0.49; 2.78)	1.24 (1.16; 1.33)
4 y	1.72 (1.61; 1.83)	0.39 (−1.32; 2.09)	1.01 80.89; 1.12)
5 y	1.43 (1.25; 1.60)	−0.86 (−3.54; 1.83)	0.77 (0.59; 0.95)
Control for drug use disorder in older sibling	1.72 (1.64; 1.79)	1.63 (0.50; 2.77)	1.01 (0.93; 1.10)
Control for parental education	1.99 (1.91; 2.07)	1.90 (0.76; 3.04)	^a^
Covariate-adjusted^b^	1.62 (1.54; 1.70)		
Sex composition of sibling pair (older-younger)			
Male–male	2.76 (2.59; 2.93)	4.01 (1.42; 6.61)	1.74 (1.55; 1.93)
Female–female	1.30 (1.18; 1.42)	−1.49 (−3.29; 0.30)	0.81 (0.68; 0.95)
Male–female	1.27 (1.16; 1.39)	1.31 (−0.51; 3.12)	0.76 (0.62, 0.89)
Female–male	2.77 (2.60; 2.95)	3.19 (0.55; 5.83)	1.64 (1.44; 1.84)
Interaction with the level of sibling genetic risk for drug use disorder	<0.0001	0.40	<0.0001
−1	0.05 (−0.05; 0.17)	2.29 (−0.62; 3.01)	−0.18 (−0.30; −0.06)
0	1.01 (0.94; 1.08)	0.50 (−0.60; 1.61)	0.77 (0.68; 0.85)
1	1.96 (1.84; 2.09)	−0.19 (−2.28; 1.89)	1.71 (1.58; 1.84)
2	2.91 (2.71; 3.12)	−0.88 (−4.44; 2.69)	2.66 (2.45; 2.87)

a Variable included in the propensity score analyses

b Based on confounder selection using lasso regression

**Figure 3.**
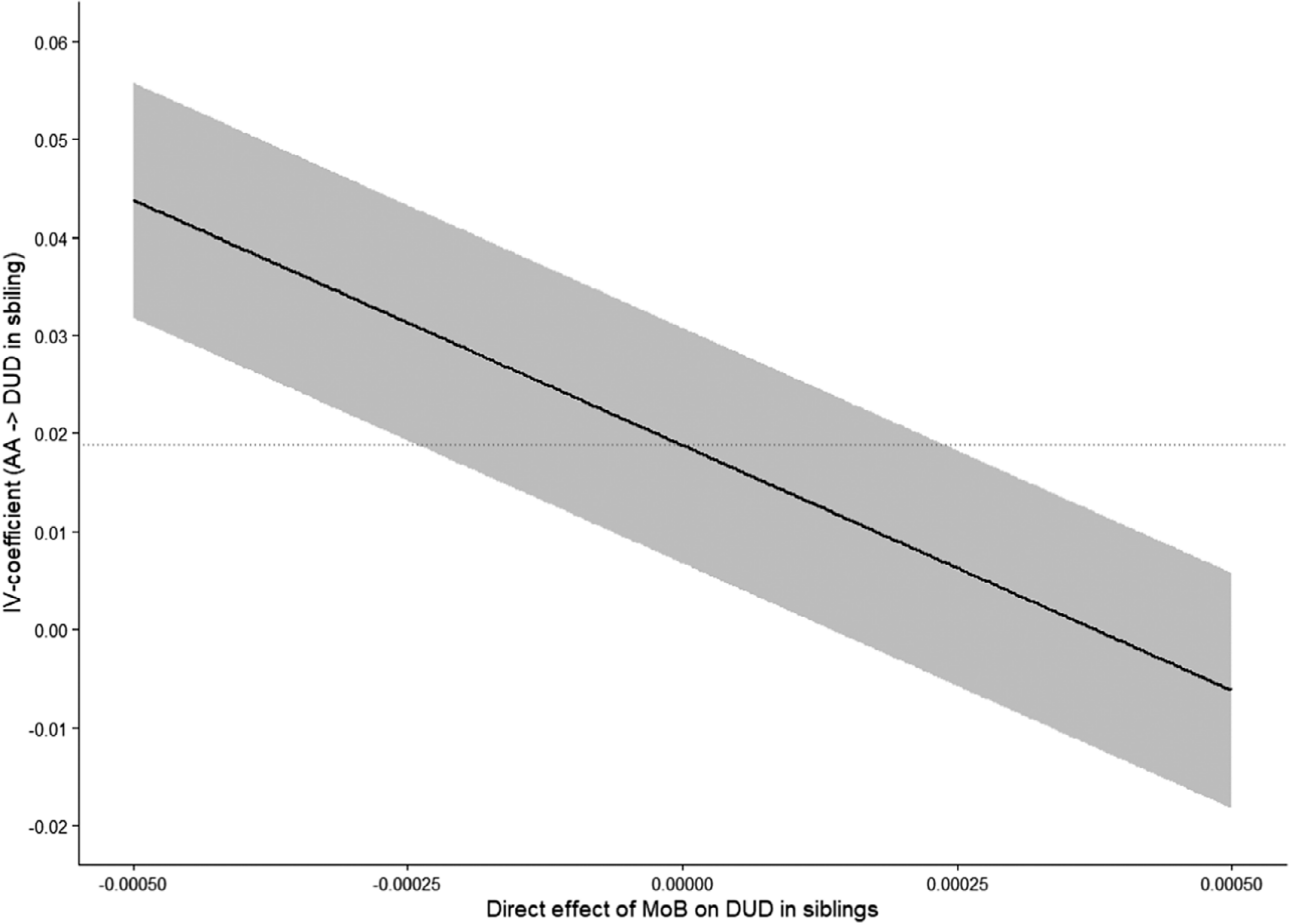
Estimated effect of academic achievement (AA) on drug use disorder (DUD) in siblings using month of birth as an instrumental variable. The solid line shows IV estimates obtained under the Conley–Hansen–Rossi union-of-confidence-intervals approach, allowing for small direct effects of MoB on the outcome. The shaded area represents the corresponding confidence intervals across assumptions about direct effects of the instrument. The horizontal dotted line indicates the reference estimate under the exclusion-restriction point assumption.

The instrument was strong, with a first-stage weak-instrument *F* = 1337.1, *p* < 0.0001. The Wu–Hausman test (*p* = 0.78) failed to reject the null hypothesis that crude OLS and IV estimates differ. Figure 3 summarizes results from the Conley–Hansen–Rossi sensitivity analysis. The baseline IV estimate corresponds to *δ* = 0, i.e. no direct effect of MoB on younger-sibling DUD. In the data, MoB was weakly associated with younger-sibling’s DUD (0.0004; 95% CI: 0.0001 to 0.0006). Scaling *δ* to this observed association, the 95% CI for the IV estimate remained positive for *δ* up to 0.0001 (25% of 0.0004). The confidence interval included zero at *δ* = 0.00015 (37.5%), and the point estimate crossed zero at approximately *δ* = 0.00035 (87.5%). Overall, these results suggest that the IV estimates remain positive under small departures from the ideal identifying assumptions, and become compatible with no effect only when relatively large direct effects are allowed.

The next analyses in Table 2 demonstrate, in all three models, that the impact of the older sib’s AA on DUD risk in the younger sib declines substantially as the age difference between the two siblings increases. We next show that, in all three models, controlling for DUD in the older sibling decreases modestly the overall effect of older sib’s AA on younger sib’s DUD risk. This analysis demonstrates that the majority of the older sibling’s effect is *not* mediated through his or her development of DUD.

To explore whether the impact of older sib’s AA could reflect parental educational status, we controlled for that in our standard and IV regressions. Very minor changes were seen in the risk predictions. For the other variables we controlled for in our IV regression, paternal criminal behavior had an impact on the IV estimate, decreasing it to 1.47 (0.29; 2.64). Nevertheless, the wide confidence intervals limit the interpretability of this estimate. Next, we examined the impact of older sib’s AA on younger sib’s DUD risk across the four possible sex pairings. Across all three models, substantially stronger effects were seen when the younger sibling was male (i.e. male–male and female–male pairs) versus when the younger sibling was female (i.e. male–female and female–female pairs.) In both causally informative models (the IV and PSA), the strongest effect was seen in male–male pairs.

Finally, we asked whether older sib AA had a stronger effect on young sib DUD risk in sibling pairs with high mean levels of FGRS_DUD_. (Being based on phenotypes of relatives, FGRS cannot meaningfully distinguish genetic risks within sibships). For both our standard and PSA regression, we saw evidence of a substantial interaction. Our IV analyses were too poorly powered to obtain meaningful results for this analysis.

As an additional robustness check, we estimated the reverse association by instrumenting younger-sibling AA with the younger sibling’s MoB and estimating its association with older-sibling DUD. The IV estimate was 2.44 (95% CI: 1.43–3.45). This finding suggests that family-level factors and/or reciprocal sibling influences may contribute to associations between siblings’ educational outcomes and DUD, and highlights the importance of sensitivity analyses when interpreting the IV estimates.

## Discussion

We sought, in these analyses, to more precisely estimate and better understand the causal impact of sibling transmission of DUD by examining the relevance of AA. To do so, we addressed four questions. First, as predicted, we found that poor AA in an older sibling was robustly associated with increased risk for DUD in a younger sibling. Given prior literature indicating that AA is transmitted within siblings (the so-called ‘spillover effect’) (Altonji et al., 2017; Hauser & Wong, 1989; Karbownik & Özek, 2023; Nicoletti & Rabe, 2019) and that AA is a strong predictor of DUD (Fothergill et al., 2008; Gauffin et al., 2013; Schulenberg et al., 1994), these results are not unexpected.

Second, the more critical question was whether we could produce results supporting possible causal effects, and the results do suggest causality. Both IV analyses, with month of birth as the instrument, and PSA suggest that a substantial majority of the association between an older sibling’s AA and the younger sibling’s DUD risk is likely causal. Importantly, the beta coefficient from the PSA results fell well within the CIs of the IV analysis, suggesting that the results replicated across methods.

Third, the prior largely descriptive literature on sibling influences on psychoactive substance use suggests these effects are strongest among male sibling pairs and siblings close in age to one another (Altonji et al., 2017; Brook et al., 1983, 1991, 1999; Kothari et al., 2014; Needle et al., 1986; Parental, 2000; Rende et al., 2005; Rowe & Gulley, 1992), and our findings align with this research. Both effects are clearly present in our IV results, albeit with large CIs, and in our PSA analyses, which had considerably greater precision in parameter estimation. In both analyses, the sibling pairs with the strongest transmissions of risk were brothers and those one year or less apart in age. When examining gender composition, the second strongest transmission was older sister to younger brother, and younger sisters did not appear as sensitive to the impact of older sibling’s deviant behaviors as younger brothers. Although few prior studies have reported outcomes according to the sex and age composition of the sibling dyad, the current results are consistent with prior findings that females are less susceptible to peer influences on drug use or delinquency than are males (Kruttschnitt, 2013; Mears, Ploeger, & Warr, 1998; Piquero, Gover, MacDonald, & Piquero, 2005).

Fourth, we sought to gain further insight into the potential causal pathway from poor AA in an older sibling to DUD in a younger sibling. In particular, we wanted to evaluate a direct pathway: poor AA in older siblings → DUD in older sibling → DUD in younger sibling. But an indirect pathway is also possible: poor AA in older siblings → general deviance in older sibling → general deviance in younger sibling → DUD in younger sibling. The key difference between these two causal schemas is whether the older sibling developing DUD is or is not a critical feature of the pathway. That is, does the DUD have to be directly transmitted between siblings or is it sufficient to transmit a general tendency toward deviant and/or antisocial behavior?. In all three of our models – raw regression, IV and PSA – controlling for older sibling DUD only modestly reduced the causal effect of older sibling low AA on younger sibling DUD risk. These results suggest that the bulk of the sibling transmission is likely mediated by a transmission of general deviance/antisocial behavior, which is nonetheless consistent with prior work demonstrating significant sibling concordance across varied antisocial outcomes (Feinberg et al., 2012). These analyses are intended to be descriptive of potential pathways and are not interpreted as formal mediation estimates.

Although sibling similarity in DUD is well established, relatively few studies have had the ability to examine how or why siblings influence each other’s behavior. Our study provides unique insight into the within-family transmission of DUD risk by identifying a modifiable environmental sibling risk factor, low AA, as increasing the risk of DUD. The results suggest that interventions designed to improve AA can reduce DUD not only in those directly targeted by such programs, but also in their siblings, thereby increasing the reach of treatment and its cost-effectiveness. These types of spillover effects have been evidenced mainly in interventions designed to improve the family environment (Caspi et al., 2018; Rowland et al., 2008), and our results also support the development of more novel therapeutic interventions that alter sibling relationships and interactions more directly (Feinberg et al., 2013).

## Limitations

These results should be interpreted in the context of five potentially significant methodological limitations. First, our assessment of DUD was limited to data in various Swedish registries. While such registry data has important advantages (e.g. no refusals or reporting biases), it cannot replicate results of interview-based assessments. Our cases are, on average, probably more severe than those meeting DSM-5 criteria for drug use disorder at interview (American Psychiatric Association, 2013). While the lifetime prevalence of DUD in Sweden is only modestly lower than that obtained in nearby Norway (Kringlen, Torgersen, & Cramer, 2001), it is considerably lower than recent epidemiologic estimates for the US (Myers et al., 2014). The validity of our definition of DUD is, however, supported by the high rates of concordance across ascertainment methods (Kendler et al., 2012) and patterns of resemblance in relatives similar to those found in personally interviewed samples (Kendler et al., 2013, 2015; Kendler, Ohlsson, Sundquist, & Sundquist, 2018). Second, causal inference from observational data is fraught with hazards and should always be treated with skepticism. Some of these concerns can be alleviated by using multiple causal inferential methods (Hammerton & Munafò, 2021; Ohlsson & Kendler, 2020), especially when based on different assumptions. This methodological approach has been termed “triangulation (Munafo & Davey-Smith, 2018).” While IV methods are a kind of natural experiment, PS analyses are one of a number of statistical approaches (Ohlsson & Kendler, 2020). The value of PS analyses increases when the data set analyzed contains many predictors of risk exposure, in this case, poor AA in adolescence. As seen in Appendix Table 3, Swedish registries contain a large number of such predictors, increasing our confidence in our PS results. Third, while sensitivity analyses allow for small direct effects of the instrument, the key identifying assumptions underlying the IV approach cannot be formally tested. Fourth, sibling influences may be bidirectional, and the shared environment could contribute to associations in both directions. Fifth, analyses adjusting for older sibling DUD are intended to explore potential pathways and should not be interpreted as formal mediation analyses.

## Conclusions

While DUD has long been known to run in families, and progress has occurred in understanding, at a potentially mechanistic level, how genetic risk factors contribute to that aggregation, understanding the basis of familial-environmental transmission is especially challenging. We previously showed that low AA in adolescence likely had a direct causal impact on the risk for subsequent DUD. In this report, we explored whether we could extrapolate that effect across siblings. We found, using two distinct causal imputation methods, that AA in the oldest sibling impacts on DUD risk in the next oldest sibling in a manner consistent with causal processes. These results were validated by showing this effect declined sharply as years between the siblings increased and was strongest in male–male pairs. Also, importantly, only a modest proportion of this effect was mediated by older siblings’ DUD. These results have clear implication for future DUD prevention trials in adolescents.

## Supporting information

10.1017/S0033291726103729.sm001 Kendler et al. supplementary material Kendler et al. supplementary material

## Data Availability

Kristina Sundquist, MD, PhD, had full access to all the data in the study and takes responsibility for the integrity of the data and the accuracy of the data analysis.

## Supplementary material

The supplementary material for this article can be found at http://doi.org/10.1017/S0033291726103729.
