# Supplementary material for: The causal impact of older siblings’ academic achievement on younger siblings’ risk for drug use disorder: instrumental variable and propensity score analyses
Source: Psychol Med. 2026 Mar 27;56:e77. doi: 10.1017/S0033291726103729 (PMC13040293; doi:10.1017/S0033291726103729)
Supplement: Kendler et al. supplementary material [file S0033291726103729sup001.docx]

Appendix

**Table 1 - Description of Registers**

The **Multi-Generation Register** is a comprehensive dataset comprising individuals who have been registered in Sweden since 1961 or who were born in 1932 or later. These individuals are referred to as index persons, and the register includes links between them and their biological parents. Currently, the register contains information on approximately 11 million index persons. The Multi-Generation Register forms part of the broader Total Population Register, with data sourced from the National Tax Board. Each year, a new version of the register is produced, incorporating new index persons born or immigrating during the year. The data from the Multi-Generation Register can be accessed for research and statistical purposes, offering valuable insights into familial and generational connections within Sweden. Further details about the content and data quality are provided in Statistics Sweden, Background Facts, Population and Welfare Statistics 2017:2, Multi-Generation Register 2016.

The **National Patient Register** (NPR) was established in the 1960s by the National Board of Health and Welfare to collect data on in-patients at public hospitals in Sweden. Initially, the register focused on psychiatric care patients and about 16% of those receiving somatic (general) care, covering only six of Sweden’s 26 county councils. In 1984, the Ministry of Health and Welfare, in collaboration with the Federation of County Councils, decided to make participation in the NPR mandatory for all county councils. By 1987, the register expanded to include all inpatient care across Sweden. Since 2001, NPR has been further broadened to cover outpatient doctor visits, including day surgery and psychiatric care, from both public and private healthcare providers. For more detailed information, you can visit the official site of the National Board of Health and Welfare: https://www.socialstyrelsen.se/en/statistics-and-data/registers/register-information/the-national-patient-register/

**Table 2 - Definition of Variables**

|  | Registers Used | Definition |
| --- | --- | --- |
| Drug Use Disorder (DUD) | The Swedish Hospital Discharge Register (coverage 1973-2018); Outpatient Care Register (national coverage 2001-2018); Primary Care Registry (Partly coverage from 1999-2018); the Swedish Drug Register (2005-2018); the Swedish Mortality Register, and the Swedish Criminal Register (1973-2018) and the Swedish Suspicion Register (1998-2018) | Drug abuse (DA) was identified in the Swedish medical and mortality registries by ICD codes (ICD8: Drug dependence (304); ICD9: Drug psychoses (292) and Drug dependence (304); ICD10: Mental and behavioral disorders due to psychoactive substance use (F10-F19), except those due to alcohol (F10) or tobacco (F17)); in the Suspicion Register by codes 3070, 5010, 5011, and 5012, that reflect crimes related to DA; and in the Crime Register by references to laws covering narcotics (law 1968:64, paragraph 1, point 6) and drug-related driving offences (law 1951:649, paragraph 4, subsection 2 and paragraph 4A, subsection 2). DA was identified in individuals (excluding those suffering from cancer) in the Prescribed Drug Register who had retrieved (in average) more than four defined daily doses a day for 12 months from either of Hypnotics and Sedatives (Anatomical Therapeutic Chemical (ATC) Classification System N05C and N05BA) or Opioids (ATC: N02A). |
| Major Depression (MD) | The Swedish Hospital Discharge Register (coverage 1973-2018); Outpatient Care Register (national coverage 2001-2018); Primary Care Registry (Partly coverage from 1999-2018) | ICD-8: 296.2, 298.0, 300.4; ICD-9: 296.2, 296.4, 298.0, 300.4; ICD-10: F32, F33. |
| Alcohol Use Disorder (AUD) | The Swedish Hospital Discharge Register (coverage 1973-2018); Outpatient Care Register (national coverage 2001-2018); Primary Care Registry (Partly coverage from 1999-2018); the Swedish Drug Register (2005-2018); the Swedish Mortality Register, and the Swedish Criminal Register (1973-2018) and the Swedish Suspicion Register (1998-2018) | Alcohol Use Disorder (AUD) was identified in the Swedish medical and mortality registries by ICD codes: ICD9: V79B, 305A, 357F, 571A-D, 425F, 535D, 291, 303, 980; ICD 10: E244, G312, G621, G721, I426, K292, K70, K852, K860, O354, T51, F10); in the Crime Register by codes 3005, 3201, which reflect crimes related to alcohol abuse; in the Suspicion Register by codes 0004, 0005 (Only those individuals with at least two alcohol-related crimes or suspicion of crimes from both Crime Register and Suspicion Register were included); in the Prescribed Drug Register by the drugs disulfiram (Anatomical Therapeutic Chemical (ATC) Classification System N07BB01), acamprosate (N07BB03), and naltrexone (N07BB04). |
| Anxiety Disorder (AD) | The Swedish Hospital Discharge Register (coverage 1973-2018); Outpatient Care Register (national coverage 2001-2018); Primary Care Registry (Partly coverage from 1999-2018) | ICD-8: 300.0, 300.2; ICD-9: 300A, 300C; ICD-10: F40, F41 |
| Bipolar Disorder (BD) | The Swedish Hospital Discharge Register (coverage 1973-2018); Outpatient Care Register (national coverage 2001-2018); Primary Care Registry (Partly coverage from 1999-2018). | ICD-8: 296.1, 296.3, 296.8, 296.9, 298.1; ICD-9: 296A, 296C, 296D, 296E, 296W, 298B; ICD-10: F30, F31 |
| ADHD | The Swedish Hospital Discharge Register (coverage 1973-2017); Outpatient Care Register (national coverage 2001-2017); Primary Care Registry (Partly coverage from 1999-2017) | ICD-9: 314; ICD-10: F90 |
| Autism spectrum disorder (ASD) | The Swedish Hospital Discharge Register (coverage 1973-2017); Outpatient Care Register (national coverage 2001-2017); Primary Care Registry (Partly coverage from 1999-2017) | ICD-9: 299; ICD-10: F840, F841, F845, F849 |
| Low education | The longitudinal integration database for health insurance and labor market studies (LISA) from 1990-2017 and The Swedish Census from 1970. | Highest achieved education measured in 1-7 levels that in turn were translated into number of years of education and then standardized with mean 0 and SD 1 by gender and year of birth. Note: In the analysis the Z-score is reversed so that higher values indicate lower education. In the registers the variable are as follows:  1-Pre-high school (7 years)  2-High School (9 years)  3-Upper Secondary School (11 years)  4-Upper Secondary School (12 years)  5-Post-secondary education (14 years)  6-Post-secondary education (17 years)  7- PhD education (21 years) |
| Unemployment status | Longitudinal integrated database for health insurance and labour market studies (LISA) | In order to be entitled to unemployment benefits the following requirements needs to be fulfilled. The individual is: unemployed (fully or partially); able to work at least 3 hours a day and 17 hours a week; registered as a jobseeker at the Employment Service; prepared to take the offer of suitable work or employment; actively seeking for a suitable job.  In our models we consider an individual as unemployed the first year the individual has more than 10 days with unemployment benefits registered |
| Social welfare recipient | Longitudinal integrated database for health insurance and labour market studies (LISA) | Social assistance is defined as financial support under the Social Services Act. You can receive support for your upkeep and for other items that you need to have a reasonable standard of living. Examples of common situations when social assistance is given: As an income supplement to low-income families; for unemployed when other unemployment assistance is not provided or is insufficient; when sickness benefits are insufficient or not provided; to those who are bound by the children in the home and cannot get childcare and therefore not can seek work. The variable is recorded at the family level, which means that all individuals in a family with social assistance will, in this report, be counted as recipients of Social welfare .  In our models we consider an individual as Social welfare recipient the first year the individual has any Social welfare benefits registered. |
| Divorce | Register of Total Population | Divorce is defined as the first divorce during our follow up period. |
| Deprivation | Longitudinal integrated database for health insurance and labour market studies (LISA), Register of Total Population | For every year an individual is registered at a specific DESO area. The DeSO areas divides Sweden into 5,983 areas and have between 700 and 2,700 inhabitants. The division considers the geographical conditions so that the boundaries follow, for example, streets, waterways and railways. Important building blocks used to create DeSO are urban areas and electoral districts. For each of the DeSO area we created a neighborhood social deprivation (SD) index based on register data for all residents in the neighborhood aged 25-64. We used deprivation indicators used by past studies to characterize neighborhood environments and then used a principal component analysis to calculate a z-score. The following four variables were included: low educational status (defined as less than 10 years of formal education); low income (from all sources, including that from interest and dividends, which was defined as less than 50% of individual median income); unemployment (defined as not employed; excluding full-time students, those completing compulsory military service, and early retirees); and social welfare assistance. We define high deprivation as above one StD from the mean value. We define deprivation as the first year of residing in a deprived area. |
| Early Retirement | Longitudinal integrated database for health insurance and labour market studies (LISA) | Early retirement is a combination of several different variables from the Swedish registers. Until year 2002, Early Retirement Pension was paid to people aged 16- 64 and granted when their working capability was deemed to be permanently reduced by at least one quarter due to medical reasons. The early retirement variable is also composed of temporary disability pensions paid to individuals whose working capability was not expected to be permanent but was expected to persist for a considerable time. From 2003, the rules for Early Retirement Pension and Temporary disability pension changed and these types of compensations were changed into sickness compensation (for individuals 30-64) and activity compensation (for individuals 19-29). The qualification rules were similar but activity compensation was supposed to be limited in time. In our models we consider an individual as early retired the first year the individual has any Early retirement benefits registered. |
| Smoking | Medical Birth Register | The register categorized maternal smoking into non-smoker, smoking 1-9, or 10 or more cigarettes per day at two time points, 3 months prior to pregnancy, and at 1st trimester. We defined smokers as individuals with any type of smoking behavior |
| Preterm birth | Medical Birth Register | 1: Before week 37; 0: Else |
| Small for Gestational Age | Medical Birth Register |  |
| Suicide Attempt (SA) | The Swedish Hospital Discharge Register (coverage 1973-2018); Outpatient Care Register (national coverage 2001-2018); Primary Care Registry (Partly coverage from 1999-2018) | IC10: X60-X84 and Y10-Y34  ICD9: E950-E959 and E980-E989  (Registrations that was follow by a suicide death within 30 days were not counted). |
| Non-affective psychosis (NAP) | The Swedish Hospital Discharge Register (coverage 1973-2018); Outpatient Care Register (national coverage 2001-2018); Primary Care Registry (Partly coverage from 1999-2018) | ICD-8: 297, 298.3, 298.9, 295.4, 295.7; ICD-9: 298E, 298W, 298X, 295E, 295H, 295W; ICD-10: F22, F23, F24, F25, F26, F27, F28, F29, F208 and codes for Schizophrenia: ICD-8: 295.1, 295.2, 2953, 295.9, 295.6; ICD-9: 295B, 295C, 295D, 295G, 295X; ICD-10: F200, F201, F202, F203, F205, F209. |
| Familial Genetic Risk Score for education (FGRSEA) | Multigenerational Register, LISA database | For all individuals in our sample we used the Multigenerational Register to identify all 2nd to 5th degree relatives. For these relatives, we used information on Number of years of education.  Number of years of education are measured in 7 different levels  1 Pre-high school < 9 years  2 High School 9 years  3 Upper Secondary School < 3 years  4 Upper Secondary School 3 years  5 Post-secondary education < 3years  6 Post-secondary education 3 years or more  7 Research education (PhD).  The educational variables is standardized with mean 0 and SD 1. For all relatives we took the mean Z-score for future calculations. For each relative we then calculated the product using two components: mean Z-score, proportion of shared genetic effects (0.003125 -0.25) with the proband. Then we average this product across all relatives to a proband. Then we corrected for the number of relatives. We multiplied the results from the previous step with a shrinkage factor. (Shrinkage factor (SF): B/(B+A/C). It produces more shrinkage if B and C are small and A is large. (A) = the variance of the z-score of the disorder across all relatives, (B) = the variance in the mean z-score across all probands,(C) = the weighted number of relatives for each proband. We standardized the risk score by year of birth and county of the proband into a z-score with mean 0 and SD 1. This was then used as the FGRS in the analyses. |
| Criminal behavior (CB) | Swedish Crime register | CB was identified by registration in the Swedish Crime register, which excludes convictions for minor crimes like traffic infractions. CB is measured using all available criminal conviction types. CB was considered a binary variable in the models. |

Table 3

Variables included in the Propensity Score Used to Predict Lower Academic Achievement in the Older Sibling

| **Variable** | Beta Coefficient |
| --- | --- |
| Low Education (M) | 0.11 (0.11; 0.11) |
| Low Education (F) | 0.10 (0.09; 0.10) |
| FGRS Low education (2-5 degree relatives) | 0.13 (0.13; 0.14) |
| Death (M) | * |
| Death (F) | 0.05 (0.02; 0.08) |
| Unemployment (F) | 0.05 (0.04; 0.06) |
| Unemployment (M) | 0.07 (0.06; 0.07) |
| Deprivation (F) | 0.03 (0.01; 0.04) |
| Deprivation (M) | 0.01 (0.00; 0.03) |
| Social Welfare (F) | 0.12 (0.10; 0.13) |
| Social Welfare (M) | 0.27 (0.26; 0.29) |
| Early Retirment (F) | 0.03 (0.01; 0.06) |
| Early Retirment (M) | 0.06 (0.04; 0.08) |
| Divorce (Parents) | 0.16 (0.15; 0.17) |
| Drug Use Disorder (F) | * |
| Drug Use Disorder (M) | * |
| Alcohol Use Disorder (F) | 0.16 (0.14; 0.17) |
| Alcohol Use Disorder (M) | 0.08 (0.04; 0.12) |
| Anxiety Disorder (F) | * |
| Anxiety Disorder (M) | * |
| Major Depression (F) | * |
| Major Depression (M) | * |
| Suicide Attempt (F) | 0.04 (0.02; 0.07) |
| Suicide Attempt (M) | 0.10 (0.07; 0.12) |
| Bipolar Disorder (F) | -0.15 (-0.22; -0.08) |
| Bipolar Disorder (M) | -0.12 8-0.18; -0.06) |
| Non-Affective Psychosis (F) | * |
| Non-Affective Psychosis (M) | * |
| Criminal Behaviour (F) | 0.20 (0.18; 0.21) |
| Criminal Behaviour (M) | 0.15 (0.13; 0.17) |
| Smoking (M) | 0.05 (0.02; 0.08) |
| Small for Gestational Age (proband) | 0.11 (0.10; 0.13) |
| Autism Spectrum Disorder (proband) | 0.25 (0.21; 0.29) |
| ADHD (proband) | 0.60 (0.58; 0.63) |
| Preterm birth (proband) | * |
| M: Mother; F: Father; * not included in the final model (variable selection based on a lasso regression. All variables are registered prior to registration of grades. | |

Table 4

Covariate‑adjusted regression estimates for the association between AA in older sibling on DUD in younger

| Covariate | Beta-coefficient |
| --- | --- |
| **AA in older Sibling** | **1.62 (1.54; 1.70)** |
| Unemployment (F) | 2.07 (1.54; 2.30) |
| Unemployment (M) | 1.72 (1.50; 1.94) |
| Early Retirement (F) | 3.80 (3.32; 4.28) |
| Early Retirement (M) | 1.92 (1.47; 2.37) |
| Divorce (Parents) | 2.10 (1.88; 2.32) |
| AUD (M) | 6.32 (5.40; 7.25) |
| SA (F) | 4.29 (3.64; 4.93) |
| SA (M) | 3.50 (2.88; 4.12) |
| ADHD | 1.83 (1.06, 2.59) |
| Smoking (M) | 4.48 (3.87; 5.09) |
| (F) – Father, (M) - Mother | |

Table 5

Associations between the instrument and measured confounders of the exposure

| Covariate  (M = Mother;  F = Father) | Association with MOB | P-value | Effect of MOB on AA while controlling for the covariate | IV effect of AA in S2 on DUD in S1 while controlling for the covariate |
| --- | --- | --- | --- | --- |
| Death (M) | -0.0002 (-0.0003; -0.0000) | 0.003 | -0.0186 (-0.0176; -0.0196) | 1.91 (0.78; 3.05) |
| Death (F) | -0.0002 (-0.0003; -0.0001) | 0.003 | -0.0186 (-0.0176; -0.0196) | 1.88 (0.74; 3.02) |
| Unemployment (F) | 0.0001 (-0.0002; 0.0005) | 0.39 | - |  |
| Unemployment (M) | 0.0000 (-0.0000; 0.0000) | 0.73 | - |  |
| Deprivation (F) | 0.0010 (0.0006; 0.0015) | <0.0001 | -0.0183 (-0.0173; -0.0193) | 1.81 (0.65; 2.97) |
| Deprivation (M) | 0.0011 (0.0007; 0.0016) | <0.0001 | -0.0183 (-0.0173; -0.0193) | 1.81 (0.65; 2.97) |
| Social Welfare (F) | 0.0005 (0.0002; 0.0008) | 0.0004 | -0.0182 (-0.0173; -0.0192) | 1.71 (0.55; 2.87) |
| Social Welfare (M) | 0.0004 (0.0001; 0.0007) | 0.0004 | -0.0183 (-0.0173; -0.0193) | 1.74 (0.58; 2.89) |
| Early Retirement (F) | 0.0000 (-0.0000; 0.0000) | 0.73 | - |  |
| Early Retirement (M) | 0.0000 (-0.0000; 0.0000) | 0.29 | - |  |
| Divorce (Parents) | 0.0000 (-0.0000; 0.0000) | 0.87 | - |  |
| Drug Use Disorder (F) | 0.0001 (0.0000; 0.0001) | 0.0208 | -0.0186 (-0.0176; -0.0196) | 1.82 (0.67; 2.96) |
| Drug Use Disorder (M) | 0.0000 (-0.0000; 0.0000) | 0.49 | - |  |
| Alcohol Use Disorder (F) | 0.0002 (0.0001; 0.0004) | 0.012 | -0.0184 (-0.0175; -0.0194) | 1.78 (0.63; 2.92) |
| Alcohol Use Disorder (M) | 0.0000 (-0.0000; 0.0000) | 0.22 | - |  |
| Anxiety Disorder (F) | 0.0000 (-0.0000; 0.0000) | 0.90 | - |  |
| Anxiety Disorder (M) | 0.0000 (-0.0000; 0.0000) | 0.17 | - |  |
| Major Depression (F) | 0.0000 (-0.0000; 0.0000) | 0.80 | - |  |
| Major Depression (M) | 0.0000 (-0.0000; 0.0000) | 0.29 | - |  |
| Suicide Attempt (F) | 0.0001 (0.0000; 0.00002) | 0.03 | - |  |
| Suicide Attempt (M) | 0.0000 (-0.0000; 0.0000) | 0.49 | - |  |
| Bipolar Disorder (F) | 0.0000 (-0.0000; 0.0000) | 0.80 | - |  |
| Bipolar Disorder (M) | 0.0000 (-0.0000; 0.0000) | 0.42 | - |  |
| Non-Affective Psychosis (F) | 0.0000 (-0.0000; 0.0000) | 0.22 | - |  |
| Non-Affective Psychosis (M) | 0.0000 (-0.0000; 0.0000) | 0.61 | - |  |
| Criminal Behaviour (F) | 0.0013 (0.0010; 0.0016) | <0.0001 | -0.0179 (-0.0169; -0.0189) | 1.47 (0.29; 2.64) |
| Criminal Behaviour (M) | 0.0003 (0.0001; 0.0005) | 0.0002 | -0.0184 (-0.0174; -0.0194) | 1.76 (0.61; 2.91) |
| Smoking (M) | 0.0010 (0.0009; 0.0011) | <0.0001 | -0.0183 (-0.0173; -0.0193) | 1.70 (0.54; 2.86) |
| Small for Gestational Age | -0.0003 (-0.0005; -0.0002) | 0.0004 | -0.0186 (-0.0176; -0.0196) | 1.88 (0.75; 3.02) |
| ASD (proband) | -0.0000 (-0.0001; 0.0000) | 0.4632 | - |  |
| ADHD (proband) | 0.0001 (0.0000; 0.0003) | 0.0219 | -0.0186 (-0.0176; -0.0196) | 1.85 (1.75; 1.95) |
| Preterm birth (proband) | 0.0000 (-0.0002; 0.0002) | 0.9049 |  |  |
|  |  |  |  |  |
